# Pressure‐Perceptive Actuators for Tactile Soft Robots and Visual Logic Devices

**DOI:** 10.1002/advs.202104270

**Published:** 2021-12-16

**Authors:** Peidi Zhou, Jian Lin, Wei Zhang, Zhiling Luo, Luzhuo Chen

**Affiliations:** ^1^ Fujian Provincial Key Laboratory of Quantum Manipulation and New Energy Materials College of Physics and Energy Fujian Normal University Fuzhou 350117 China; ^2^ Fujian Provincial Collaborative Innovation Center for Advanced High‐Field Superconducting Materials and Engineering Fuzhou 350117 China; ^3^ Fujian Provincial Engineering Technology Research Center of Solar Energy Conversion and Energy Storage Fuzhou 350117 China

**Keywords:** carbon nanotubes, logic devices, pressure‐perceptive actuators, sensors, silk

## Abstract

Soft actuators with sensing capabilities are important in intelligent robots and human–computer interactions. However, present perceptive actuating systems rely on the integration of multiple functional units with complex circuit design. Here, a new‐type pressure‐perceptive actuator is reported, which integrates functions of sensing, actuating, and decision making at material level without complex combination. The actuator is composed of an actuating unit and a pressure‐sensing unit, both of which are fabricated by carbon nanotube (CNT), silk, and polymer composite. On the one hand, the actuating unit can be driven by low voltages (<13 V), owing to a Joule‐heating effect. On the other hand, the current passing the pressure‐sensing unit can be controlled by tactile pressure. In the integrated actuator, it is able to control the deformation amplitude of actuating unit by applying different pressures on the pressure‐sensing unit. A portable tactile‐activated gripper is fabricated to operate an object through pressure control, demonstrating its application in tactile soft robots. Finally, three visual logic gates (AND, OR, and NOT) are proposed, which convert “tactile” inputs into “visible” deformation outputs, using the CNT‐silk‐based material for sensing and actuating in the decision‐making process. This study provides a new path for intelligent soft robots and new‐generation logic devices.

## Graphical abstract

A pressure‐perceptive actuator is proposed, which is composed of an actuating unit and a pressure‐sensing unit. It is able to control the deformation amplitude of actuating unit by tactile pressure through the pressure‐sensing unit. A portable tactile‐activated gripper is fabricated to demonstrate the application in tactile soft robots. Finally, three visual logic gates (AND, OR, and NOT) are proposed.

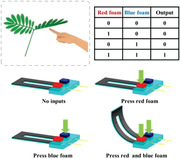

## 1 Introduction

Soft actuating materials are important in the development of intelligent robots. The elastic modulus of soft robots is similar to that of soft tissue in biological systems, which makes soft robots safer and more robust than rigid robots.^[^ ^1^, ^2^ ^]^ In order to obtain autonomy feedback of intelligent soft robots, it is crucial to develop a soft actuating system with sensing/perception capabilities. Current soft actuating and sensing systems can be classified into two categories. By integrating the actuating material with the sensor, one kind of actuating system recognizes the signals from the sensor and responds to the signals accordingly,^[^ ^3^, ^4^, ^5^ ^]^ which makes the actuating system inevitably require the intervention of central processor and lead to complexity of intelligent robot composition and energy loss. Another kind of actuating and sensing system is based on actuating materials with sensing/perception functions,^[^ ^6^, ^7^, ^8^ ^]^ which can respond to external stimuli such as light,^[^ ^9^, ^10^ ^]^ electricity,^[^ ^11^, ^12^ ^]^ temperature,^[^ ^13^, ^14^ ^]^ and humidity.^[^ ^15^, ^16^ ^]^ However, these actuating and sensing systems lack the response to mechanical stimuli, which limits their application in bionic systems and human–computer interactions.

**Figure 1:**
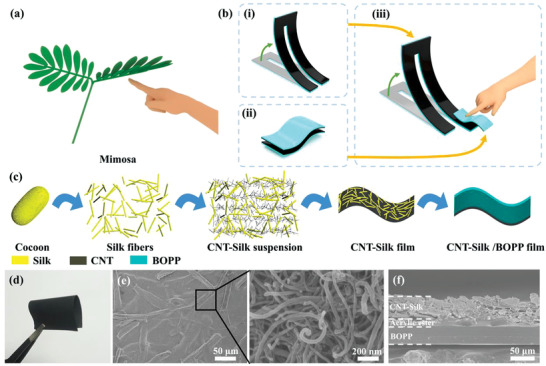
a) Schematic diagram of Mimosa stimulated by mechanical touch. b) Schematic diagrams showing the components of pressure‐perceptive actuator: i) electrothermal actuating unit, ii) pressure‐sensing unit, and iii) pressure‐perceptive actuator. c) Schematic diagram showing the fabrication of CNT–Silk/BOPP composite film. d) Optical photo showing the flexibility of CNT–Silk composite film. e) SEM images of the surface morphology of the CNT–Silk composite film. f) Cross‐sectional SEM image of the CNT–Silk/BOPP composite film.

Mechanical stimuli are very common in our daily life. Touch interaction is currently one of the most important ways of human–computer interaction, and is most commonly used in cell phones. This type of interaction sets users free from superfluous input devices (such as mouse and keyboard) and simplifies the human–computer interaction, which may further appear in intelligent robots, smart homes, and virtual reality. Besides, the phenomenon of responding to mechanical stimuli is widespread in natural organisms. Take Mimosa as an example, when it is touched by a finger, its leaves will close, which is a deformation response to external mechanical stimuli (**Figure** **1**a). The Mimosa does not use a central processor when processing external mechanical stimulus signals, which simplifies the information processing process. On the contrary, most of the artificial fabricated sensors can only perceive mechanical stimulus changes, and the signal processing and conversion by a central processor are necessary if visual information or behavioral feedback is desired.

The biological proprioceptive phenomena have a well‐informed inspiration for us to build intelligent actuating systems. Meanwhile, compared to the feedback of electrical signals, mechanical feedback is more probable to provide solutions to real‐life problems. For example, the organism will instinctively take cover when it senses outside threat. Currently, only a few actuating systems are capable of providing mechanical feedback to mechanical stimuli. He et al. proposed an artificial somatic reflex system that enabled electrochemical actuators to respond to tactile pressure stimuli, and also integrated it into a robot to mimic the grasp reflex of an infant.^[^ ^17^ ^]^ The system allows the collection of physical signals from the environment and generates feedback motions, and provides a promising direction for subsequent research on the actuation and control of intelligent robots. However, the above actuating system has some shortcomings. First, the system consists of three different parts, which are based on different materials, and the integration of three parts requires a relatively complex circuit design. Second, the response behavior cannot be stopped once enough stimulus is given, which is unfavorable for industrial production that may require an “emergency stop”. Finally, only when the voltage polarity of actuation circuit is reversed, the posture in the system can be released, which has some limitations for its further application.

Here, we propose a pressure‐perceptive actuator, which consists of only two parts: an electrothermal actuating unit and a pressure‐sensing unit. Both of them are composed of carbon nanotube (CNT)‐silk composite and biaxially oriented polypropylene (BOPP) film. The CNT‐based actuating unit is capable of deformation when stimulated by external electricity, which mimics the movement of muscles (Figure 1bi). The pressure‐sensing unit effectively responds to pressure and controls the amount of current in the circuit, which mimics the perception of mechanical stimulus by biological tissues (Figure 1bii). The ingenious combination of these two units allows the deformation of actuator to be controlled by mechanical stimulus (Figure 1biii). To prove this point, a tactile‐activated gripper is fabricated to grasp and release objects by controlling the pressure‐sensing unit. The actuators in gripper can be driven by a low voltage provided by an integrated battery, so the gripper is portable and can be operated without an additional power supply. Finally, three visual logic gates (AND, OR, and NOT) are constructed through advanced structural design. The deformation of actuating units in logic gates depends on the input tactile signal and the material itself, which can redistribute the electrical energy according to the tactile signal, avoiding the use of a central processor. The visual logic gates achieve the transformation of “tactile” signals to “visual” signals (deformation of actuating unit). We believe that this study will provide new ideas and directions for intelligent soft robotic systems with autonomous control based on nanomaterials.

## 2 Results and Discussion

### 2.1 Characterizations of CNT–Silk/BOPP Film

The CNT–Silk/BOPP film plays an important role, which is used for actuators or pressure sensors. Actuating and sensing functions can also be perfectly combined in one device. The fabrication method of CNT–Silk/BOPP film is shown in Figure 1c. Fabrication details are described in the “Materials and Methods” section in Note S1 (Supporting Information). Silk fiber produced from cocoons is composed of fibrin and sericin that surrounds fibrin fibers. The silk fiber after removing the gelatinous sericin was used as a skeleton to obtain a self‐supporting CNT–Silk composite film, since normal commercial CNTs are difficult to form a self‐supporting film. At the same time, the CNT–Silk film has good flexibility (Figure 1d). Scanning electron microscope (SEM) images of the CNT–Silk film surface are shown in Figure 1e. The silk fibers are evenly distributed to form a skeleton (left panel of Figure 1e). Meanwhile, CNTs are uniformly on the surface of silk fiber or filled in the pores formed by the silk fibers (right panel of Figure 1e and Figure S1 in Supporting Information). According to the Raman spectra of pure CNT and CNT–Silk composite film (Figure S2a in the Supporting Information), the characteristic D and G bands of CNT are marked at about 1346 and 1582 cm^−1^, which are in accordance with previous reports.^[^ ^18^, ^19^ ^]^ Meanwhile, as shown in Figure S2b (Supporting Information), the X‐ray diffraction (XRD) patterns of pure silk and CNT–Silk composite have the same peak positions at about 21°.^[^ ^20^ ^]^ And intrinsic peaks of CNT can also be clearly seen in the XRD patterns of CNT and CNT–Silk composite.^[^ ^21^, ^22^ ^]^ It can be found that the crystalline structure of the CNT and silk fiber did not degrade during the fabrication process. Finally, a flexible BOPP film coated with acrylic ester was flatly attached to the surface of CNT–Silk film to obtain a CNT–Silk/BOPP composite material. The SEM image in Figure 1f illustrates the cross‐sectional microstructure of the CNT–Silk/BOPP film. It can be seen that the CNT–Silk layer and BOPP layer are tightly bonded together by acrylic ester. Due to the introducing of CNTs, the CNT–Silk/BOPP film has good electrical conductivity (*R*_□_ = 21.8 Ω) and good flexibility. The relative resistance change (Δ*R*/*R*_0_) of the CNT–Silk and CNT–Silk/BOPP films with different radii (*r*) were tested, where *R*_0_ is the initial resistance before bending and Δ*R* is the resistance change after bending. As shown in Figure S3 (Supporting Information), both the CNT–Silk and CNT–Silk/BOPP films were bendable, and their resistances showed no noticeable dependence on the bending curvature. These properties are essential for making soft electronic devices.

### 2.2 Actuation Performance of CNT–Silk/BOPP Actuator

**Figure 2:**
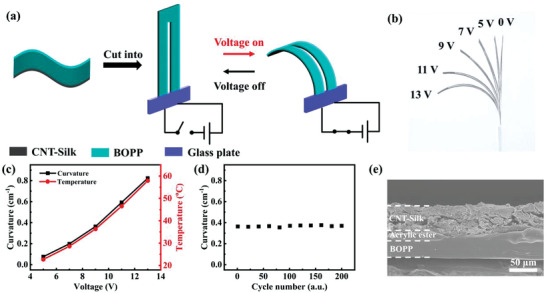
a) Schematic illustration of the electrothermal actuation process. b) Optical photo of the CNT–Silk/BOPP actuator driven by different voltages for 5 s, respectively. c) Maximal bending curvature (black solid curve) and maximal temperature (red solid curve) of the CNT–Silk/BOPP actuator as a function of voltage. d) Repeatability test on the actuation performance of the CNT–Silk/BOPP actuator driven by a voltage of 9 V for 200 cycles. e) Cross‐sectional SEM image of the CNT–Silk/BOPP actuator after the repeatability test.

The CNT–Silk/BOPP film is first used to fabricate actuators. As shown in **Figure** **2**a, the prepared actuator was an electrothermal actuator with a double‐layer structure. The fabrication details of the actuator are described in the “Materials and Methods” section in Note S1 (Supporting Information). The actuator was cut into U‐shape and the copper electrodes were embedded in two ends of the actuator. One layer of the actuator is the BOPP film with a large coefficient of thermal expansion (CTE) (137 ppm K^−1^).^[^ ^23^, ^24^ ^]^ Meanwhile, CNT has a small CTE (16–26 ppm K^−1^).^[^ ^25^, ^26^ ^]^ And the silk expands by absorbing water,^[^ ^20^, ^27^ ^]^ thus it loses water and shrinks when heated. When the CNT–Silk/BOPP actuator is driven by a voltage, the highly conductive CNT–Silk layer is used as an electrothermal heating component. The kinetic energy of electrons is converted to thermal energy due to the resistance of CNT–Silk layer (conductor) to the electrons flow, which is called the Joule‐heating effect.^[^ ^28^ ^]^ Thus, the CNT–Silk layer converts electrical energy into thermal energy, and the temperature of actuator rises. Because the CTEs of two layers in the actuator are different, the expansion of BOPP layer will be larger than that of CNT–Silk layer when the temperature increases. Meanwhile, the CNT–Silk layer and BOPP layer are coupled tightly, so the generated internal stress will make the actuator bend toward the CNT–Silk side.

The actuation performance of CNT–Silk/BOPP actuator was studied. Since the silk is hydrophilic, the environmental relative humidity (RH) will affect the actuation performance of actuator. A detailed discussion is provided in Note S2 and Figure S4 (Supporting Information). To eliminate the influence of RH changes on the actuator, the RH during the testing was kept the same as that during the fabrication of samples. The dimensions of the U‐shape CNT–Silk/BOPP actuator are shown in Figure S5 (Supporting Information). The optical photo (Figure 2b) clearly shows the bending deformation of the actuator under different driving voltages for 5 s, respectively. When no voltage was applied (0 V), the actuator was in flat state. When the driving voltage increased, the bending angle of the actuator became larger. The calculation of bending curvature is explained in Note S3 and Figure S6 in the Supporting Information. The variations of bending curvature under different voltages are shown in Figure S7a (Supporting Information). And the temperature of actuator was also measured in real time (Figure S7b in the Supporting Information). When the voltage was turned on, the temperature started to rise and the curvature began to change. When the voltage was turned off, the temperature of the actuator began to drop immediately, and the deformation of actuator recovered immediately. It can be seen that a higher driving voltage resulted in a higher temperature and larger deformation of the actuator (Figure 2c), which reveals that the actuation is caused by a Joule‐heating effect. When the driving voltage increased to 13 V, the temperature of actuator reached 58 °C and the bending curvature reached 0.82 cm^−1^. The mechanical properties of the actuator were also studied. As shown in Figure S8a (Supporting Information), the Young's moduli of CNT–Silk film and BOPP film were 485 and 1258 MPa, respectively. Figure S8b (Supporting Information) shows the blocking force of actuator increased with driving voltages. The inset diagram shows the test schematic. The test details are described in the “Materials and Methods” section in Note S1 (Supporting Information). When the driving voltage was 13 V, the blocking force reached 0.66 mN. The force density was 4.2 (blocking force divided by the weight), which indicated that the actuator had good mechanical output capability. Besides, the repeatability performance of the actuator was tested. As shown in Figure 2d, the bending performance of the actuator did not change obviously during 200 cycles. The cross‐sectional SEM image of the actuator after the test also showed that there was no delamination between the two layers in actuator after the repeatability test (Figure 2e), indicating the good stability of the CNT–Silk/BOPP actuator.

### 2.3 Pressure‐Perceptive Performance of the CNT–Silk/BOPP Pressure Sensor

**Figure 3:**
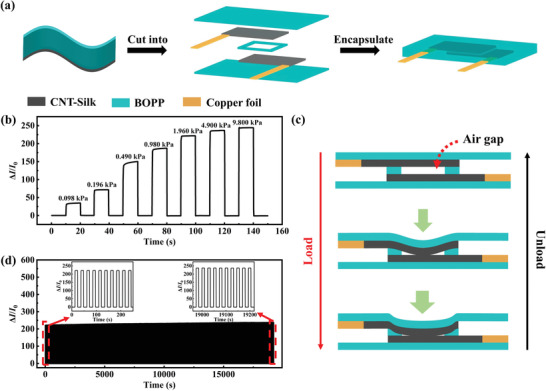
a) Schematic diagram showing the fabrication of CNT–Silk/BOPP pressure sensor. b) Current change of the pressure sensor under different pressures. c) Schematic diagram illustrating the sensing mechanism of the pressure sensor. d) Repeatability test of the sensing performance of the pressure sensor for 800 cycles under pressure of 1.960 kPa.

Because the CNTs are introduced into the device, the CNT–Silk film has good electrical conductivity. Also, there are many microstructures on the surface of CNT–Silk film (Figure 1e), which significantly increases the surface roughness. The above characteristics make the CNT–Silk film as potential sensing materials. The manufacturing process of the pressure sensor is shown in **Figure** **3**a. The dimensions of pressure sensor are shown in Figure S9a (Supporting Information). The pressure sensor had a sandwich structure. Two CNT–Silk/BOPP films were placed face to face, and a BOPP film with a loop structure was placed between them. The optical photo of prepared pressure sensor is shown in Figure S9b (Supporting Information).

Next, the sensing performance of the CNT–Silk/BOPP pressure sensor was studied. The electrical current response of the sensor under different pressures is shown in Figure 3b. It can be found that the sensor can achieve a wide pressure range (0.098–9.8 kPa), which can match the pressure when a human finger touches or presses. At the same time, when the pressure is applied, a stable current pattern can be obtained. Sensitivity is a paramount parameter for evaluating the performance of a pressure sensor and it is defined as *S* = (Δ*I*/*I*_0_)/Δ*P*, where Δ*I* represents the current change in a certain pressure range, *I*_0_ represents the primal current without pressure, and Δ*P* represents the pressure change. As shown in Figure S10 (Supporting Information), the sensitivity of CNT–Silk/BOPP pressure sensor was 188.9 kPa^−1^ below 0.98 kPa, and it was 5.4 kPa^−1^ in the pressure range of 0.98–9.8 kPa. The pressure sensor has a high sensitivity, which gradually decreases as the pressure increases. Figure 3c shows a schematic diagram showing the sectional view of the pressure sensor before and after applying pressure. When no pressure is applied, there is an air gap between the CNT–Silk films. When a low pressure is applied to the sensor, the air gap between CNT–Silk films becomes thinner, and the contact area will greatly increase. Then, the resistance of pressure sensor decreases rapidly (the current increases rapidly). However, when the pressure reaches a certain level, the air gap between the CNT–Silk films disappears, and the resistance change will be smaller (the current change becomes slower). Therefore, the CNT–Silk/BOPP pressure sensor has a high sensitivity in the low‐pressure range (<0.98 kPa) and a low sensitivity in the high‐pressure range (0.98–9.8 kPa). Figure 3d shows the repeatability test results of the CNT–Silk/BOPP pressure sensor for 800 cycles under the pressure of 1.960 kPa. It shows that the pressure sensor has good repeatability. The magnified insets of Figure 3d clearly show that there is no significant degradation during the entire cycling test, which means that the air gap can still exist stably under repeated pressure and does not cause a gradual decrease to the sensing performance. Compared with previous reported CNT‐based pressure sensors, the CNT–Silk/BOPP pressure sensor proposed in our study has the advantage in sensitivity. Detailed comparisons are listed in Table S1 (Supporting Information).^[^ ^29^, ^30^, ^31^, ^32^, ^33^, ^34^, ^35^, ^36^, ^37^, ^38^ ^]^ These results indicate that CNT–Silk/BOPP composite films can be used in pressure‐sensing devices for further applications.

### 2.4 Tactile‐Activated Gripper

**Figure 4:**
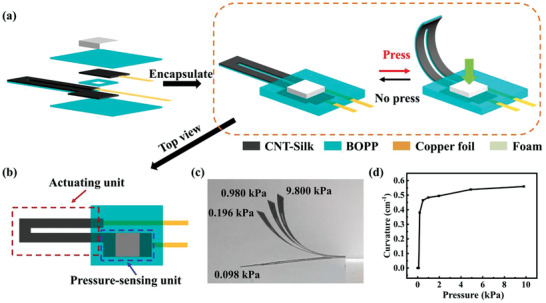
a) Schematic diagram of the CNT–Silk/BOPP actuator integrated with an actuating unit and a pressure‐sensing unit. b) Schematic diagram of the top view of the CNT–Silk/BOPP actuator integrated with an actuating unit and a pressure‐sensing unit. c) Optical photo of the CNT–Silk/BOPP actuator under different pressures for 5 s (applied voltage: 13 V). d) Maximal bending curvature of the CNT–Silk/BOPP actuator as a function of pressure (applied voltage: 13 V).

In previous sections, we have introduced two devices based on CNT–Silk film: the electrothermal actuator and the pressure sensor. The actuator is driven by electrical stimulation, and the sensor can adjust the output current by changing pressure. The sensor can be designed as a pressure‐sensing unit to control the amplitude of actuator. Therefore, an actuator integrated with a pressure sensor (pressure‐sensing unit) was fabricated. The detailed fabrication process is described in the “Materials and Methods” section of Note S1 (Supporting Information). The composition of the actuator is shown in the left panel of **Figure** **4**a. It consists of two parts, the actuating unit (red dotted frame) and the pressure‐sensing unit (blue dotted frame) (Figure 4b). In order to use the pressure‐sensing unit more conveniently, a foam cube was placed directly on it. First, the foam cube allows the user to quickly identify the position of the pressure‐sensing unit in the integrated device. Second, the protruding foam cube can make the pressing more comfortable.

Initially, a driving voltage of 13 V was applied to the integrated actuator through the pressure‐sensing unit. As shown in the right panel of Figure 4a, when no pressure was applied, there was almost no current passing the pressure‐sensing unit, and the actuator could not be driven. When a pressure was applied, the current through the pressure‐sensing unit would change accordingly, and the actuator would deform. As shown in Figures 4c,d, when no pressure was applied or the applied pressure was low (<0.098 kPa), the actuator did not bend, indicating that the current in the circuit was insufficient to drive the actuator. When the applied pressure became higher, the actuator began to bend. The reason is that the current crossing the actuator increases with the applied increasing pressure (same as the previous sensing part), resulting in the bending amplitude enhancement of the actuator. When the pressure was 9.8 kPa, the bending curvature of actuator was up to 0.56 cm^−1^. The above results indicate that we can control the deformation amplitude of actuator through pressure. In daily life, human finger is often used to operate objects. Although the finger cannot apply pressure to objects with the same precision as a machine, it still can apply different mechanical stimuli to objects. To further confirm this idea, we use fingers to touch or press the foam cube on the actuator. Initially, when no pressure was applied, the actuator did not bend (Figure S11a in Supporting Information). The deformation of actuator was different when the foam cube was gently and heavily pressed by the finger (Figure S11b,c in the Supporting Information). These results show that we are able to integrate the above CNT–Silk/BOPP‐based actuators and sensors to prepare a new type of pressure‐perceptive actuator.

**Figure 5:**
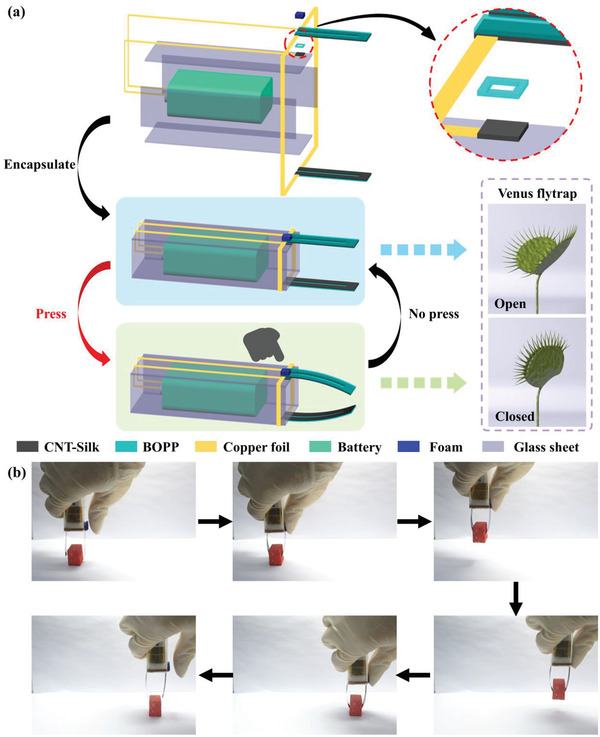
a) Schematic diagram of the tactile‐activated gripper based on CNT–Silk/BOPP actuators. b) Optical photos of the tactile‐activated gripper grasping and moving an object.

The pressure‐perceptive actuator integrates the functions of actuator and pressure sensor in one device. These two functions are not simply superimposed. The combination changes the original driving method, and controls the actuator in a more convenient and intelligent way. In nature, Venus flytrap is the most famous carnivorous plant, whose leaves have many small antennae. Once an object contacts the antennae of Venus flytrap, its leaves will automatically close and trap extraneous objects. Inspired by the Venus flytrap, a tactile‐activated gripper was designed based on the features CNT–Silk/BOPP composite. The detailed fabrication process is described in the “Materials and Methods” section in Note S1 (Supporting Information). As shown in the upper panel of **Figure** **5**a, the tactile‐activated gripper mainly consisted of three parts: CNT–Silk/BOPP actuating unit, CNT–Silk/BOPP pressure‐sensing unit, and battery. A blue foam cube was placed on the pressure‐sensing unit. Figure S12a,b in the Supporting Information shows the optical photos of tactile‐activated gripper. The pressure‐sensing unit of the tactile‐activated gripper simulates the antennae part of flytrap and converts tactile signals into electrical signals. The actuating unit of the tactile‐activated gripper can simulate the leaf part of the flytrap, which can deform after receiving the signal. The CNT–Silk/BOPP actuator can be driven by low voltages (5–13 V). To prove the tactile‐activated gripper is portable and easy to operate, a battery (9 V) was integrated in the gripper as the power source. In this way, the tactile‐activated gripper can operate without the need for an external power supply.

The lower panel of Figure 5a shows the working mechanism of tactile‐activated gripper. When no pressure was applied to the pressure‐sensing unit of gripper, the resistance of the sensing unit was very large, so the circuit was in an “open‐circuit” state (Figure S12c, Supporting Information). When a certain pressure was applied to the pressure‐sensing unit of gripper (pressing the foam cube), the resistance of sensing unit dropped sharply, and the circuit was in the “closed circuit” state (Figure S12d, Supporting Information). At the same time, the actuating units deformed due to the electrothermal actuation. Once the pressure was removed (releasing the foam cube), the circuit returned to the “open‐circuit” state and the actuating units would return to their original state. The gripper can realize a real‐time response to pressure. The optical photos in Figure 5b show that when the foam cube on the tactile‐activated gripper was pressed by a finger, the gripper could grasp and move an object that was 2.3 times heavier than the weight of an actuating unit. The corresponding video is shown in Movie S1 (Supporting Information). The above results show that the tactile‐activated gripper can simulate the response of a Venus flytrap when it is stimulated by external pressure (capturing objects), and such response does not require the use of a central processing unit.

### 2.5 Visual Logic Gates Based on CNT–Silk/BOPP Composites

The performances of CNT–Silk/BOPP composite enable us to further design novel logic devices, in which simple logic decisions can be achieved without using a central processor. We demonstrate the above point and propose “visual” logic gates based on CNT–Silk/BOPP composites.

#### 2.5.1 AND Gate

**Figure 6:**
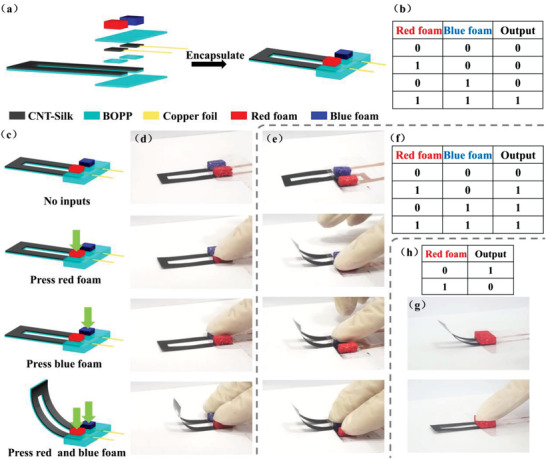
a) Schematic diagram showing the fabrication of a visual “AND gate”. b) Truth table of the visual “AND gate”. c) Schematic diagram showing the mechanism of the visual “AND gate”. d) Optical photos showing the visual “AND gate”. e) Optical photos showing the visual “OR gate”. f) Truth table of the visual “OR gate”. g) Optical photos showing the visual “NOT gate”. h) Truth table of the visual “NOT gate”.

As shown in **Figure** **6**a, a visual logic device that implements a function similar to an AND gate (Figure 6b) was designed. The device consists of two input pressure‐sensing units (red foam and blue foam) and one output actuating unit. The two input pressure‐sensing units are connected to two ends of the actuating unit respectively. A voltage of 13 V was applied to the device. According to the language of binary logic, applying pressure to the foam cube on pressure‐sensing unit is defined as input signal “1”, and no applying pressure to the foam cube is defined as input signal “0”. The actuating unit responds to the input by whether it is deformed. The deformation state of the actuating unit is defined as output signal “1”. and the no deformation state of it is defined as output signal “0”. As shown in Figures 6c,d, when no pressure was applied to red and blue foam cubes, the input signals were “0, 0”, and the circuit was in an “open‐circuit” state. The actuating unit did not deform, and the output signal was “0”. When the pressure was applied to only one of the foam cubes (red or blue foam), the input signals were “1, 0” or “0, 1”, and the circuit was still in an “open‐circuit” state. The actuating unit did not deform and the output signal was “0”. When the pressure was applied to both red and blue foam cubes, the input signals were “1, 1”, and the circuit was in the “closed circuit” state. The actuating unit deformed and the output signal was “1”. The circuit schematic diagrams in different states are shown in Figure S13 (Supporting Information). In this device, the input signals are pressure, and the output signal is the deformation of actuator. This output is mechanical energy and a “visual” signal that can be observed, realizing a “visual” AND gate.

#### 2.5.2 OR Gate

Similarly, we have designed a visual OR gate. The main difference of the device structure in the OR gate compared to that of the AND gate is that two pressure‐sensing units are in parallel and connected to only one end of the actuating unit. More detailed descriptions are provided in Note S4 and Figure S14a,b in the Supporting Information. As shown in Figure 6e,f, when no pressure was applied to red and blue foam cubes, the input signals were “0, 0”, the actuating unit did not deform and the output signal was “0”. When the pressure was applied to one of the foam cubes (red foam or blue foam) or even both of the foam cubes, the input signals were “1, 0”, “0, 1” or “1, 1”. For all the above three input signals, the actuator was deformed and the output signal was “1”. The circuit diagrams are in Figure S14c (Supporting Information).

#### 2.5.3 NOT Gate

A visual NOT gate based on the CNT–Silk/BOPP composite was also fabricated. The main difference of the device structure in the NOT gate is that only one pressure‐sensing unit is connected to two ends of the actuating unit. Therefore, pressing on the foam cube will result in a “short‐circuit” state. More detailed descriptions are provided in Note S4 and Figures S15a,b in the Supporting Information. As shown in Figures 6g,h, when no pressure was applied to the foam cube, the input signal was “0”, the actuating unit deformed and the output signal was “1”. When the pressure was applied to the foam cube, the input signal was “1”. As most current did not pass through the actuating unit due to the “short‐circuit” state, the actuating unit did not deform and the output signal was “0” The circuit diagrams are in Figure S15c (Supporting Information).

The logic responses in our visual logic gates are built entirely by soft materials locally, which are convenient and comfortable for human use, eliminating the need for traditional semiconductor‐based logic elements and central processors. Through unified raw materials and ingenious preparation steps, electrical signals and “tactile” signals are converted into mechanical energy and “visual” signals. This feedback method can not only output “visual” signals but also give behavior feedback, which is different from traditional touch‐interaction systems. Due to behavioral feedback, it is very friendly for those who are color blind or who need to receive information by touch. At the same time, the output signal can be further utilized. Motion feedback can be directly used to grasp or move objects (similar to the tactile‐activated gripper described in the previous section), and different input signals determine whether to perform the desired action. Alternatively, behavior feedback can also be used in the circuit. The actuating unit can be used as a switch unit in the circuit, and the distribution of current in the circuit can be determined by the input signal indirectly. Furthermore, the current in the logic device itself can also be used as an output signal. Take a concept as an example, a visual logic gate can be connected in parallel to another circuit that needs to be controlled. The input signal can directly determine the magnitude of current in that circuit, and users can get direct feedback from the actuating unit of visual logic gate. In short, the logic feedback device based on the material itself can be further developed and utilized in the future.

## 3 Conclusion

In summary, we first propose two kinds of intelligent devices based on the CNT–Silk/BOPP composite film: the electrothermal actuator and the pressure sensor. These two devices show excellent performances in actuating and pressure‐sensing. Later, we integrate the pressure sensor into the actuator as a pressure‐sensing unit. The integrated device can simulate the action of the Venus flytrap that closes its leaves when stimulated by external pressure. The electrical current can be adjusted through the pressure‐sensing unit by changing the tactile signal, which also passes through the actuating unit, resulting in a visible actuating deformation. Finally, we propose a new way to make decisions that redistribute the current in the circuit by “tactile” signal and give feedback through “visual” motions. We have implemented the functions of logic AND gate, OR gate, and NOT gate. The logical decision depends on the material itself and the composition of circuit, which can occur locally without a central processor. This way of making decisions offer a new kind of interactive mode and a new way of local control. Finally, we hope that this new‐type pressure‐perspective actuator will open a new path to the development of e‐skins, soft robots, and intelligent electronics.

## Conflict of Interest

The authors declare no conflict of interest.

## Supporting information

Supporting Information

Supplemental Movie 1

## Data Availability

The data that support the findings of this study are available from the corresponding author upon reasonable request.
