# Supplementary material for: Pressure‐Perceptive Actuators for Tactile Soft Robots and Visual Logic Devices
Source: Adv Sci (Weinh). 2021 Dec 16;9(5):2104270. doi: 10.1002/advs.202104270 (PMC8844481; doi:10.1002/advs.202104270)
Supplement: Supplementary file 1 — Supporting Information [file ADVS-9-2104270-s001.pdf]

ADVANCED SCIENCE
Open Access

# Supporting Information

for *Adv. Sci.*, DOI: 10.1002/advs.202104270

*Pressure-Perceptive Actuators for Tactile Soft Robots and Visual Logic Devices*

*Peidi Zhou, Jian Lin, Wei Zhang, Zhiling Luo, and Luzhuo Chen**

# Supporting Information

**Pressure-Perceptive Actuators for Tactile Soft Robots and Visual Logic Devices**

*Peidi Zhou, Jian Lin, Wei Zhang, Zhiling Luo, and Luzhuo Chen**

P. Zhou, J. Lin, Dr. W. Zhang, Dr. Z. Luo, Prof. L. Chen
Fujian Provincial Key Laboratory of Quantum Manipulation and New Energy Materials
College of Physics and Energy, Fujian Normal University, Fuzhou 350117, China
E-mail: ChenLZ@fjnu.edu.cn
P. Zhou, J. Lin, Dr. W. Zhang, Dr. Z. Luo, Prof. L. Chen
Fujian Provincial Collaborative Innovation Center for Advanced High-Field Superconducting Materials and Engineering, Fuzhou 350117, China
P. Zhou, J. Lin, Dr. W. Zhang, Dr. Z. Luo, Prof. L. Chen
Fujian Provincial Engineering Technology Research Center of Solar Energy Conversion and Energy Storage, Fuzhou 350117, China

**Note S1**

## Materials and Methods

### Materials

Cocoons were purchased from the farm. CNTs (functionalized with carboxyl) were purchased from Chengdu Organic Chemicals Co. Ltd., Chinese Academy of Sciences. BOPP films (coated with acrylic ester) were commercial products.

### Fabrication of CNT-Silk/BOPP Film

First, the cocoons were cut into small pieces with dimensions of about 20 mm × 20 mm, and then these small pieces of cocoons were boiled in $Na_2CO_3$ ($0.05 mol·L^{-1}$) aqueous solution for 2 hours. Then, the solution was infiltrated under evacuation followed by rinsing thoroughly with deionized water to remove the glue-like sericin proteins. The silk fibers were collected and dried at 65 °C. Second, the CNT (25 mg) and silk (20 mg) were dispersed in deionized water (30 mL) with the aid of ultrasonic. Then, the CNT-Silk suspension was obtained. Third, the CNT-Silk film was obtained by infiltration of the CNT-Silk suspension and dried at 65 °C. The thickness of CNT-Silk film was ~ 55 μm. Finally, a commercial BOPP film coated with acrylic ester was attached to the CNT-Silk film to form a CNT-Silk/BOPP film. The thickness of CNT-Silk/BOPP film was ~ 95 μm. It should be noted that if the content of silk is decreased, a uniform and self-supporting CNT-Silk film cannot be obtained by vacuum filtration. And if the content of silk is increased, the resistance of fabricated CNT-Silk film will be larger, which will increase the driving voltage of the CNT-Silk/BOPP actuators. In this study, the samples were fabricated and tested under RH of 55±5%.

### Fabrication of CNT-Silk/BOPP Actuator

The electrothermal actuator is U-shaped. The CNT-Silk/BOPP film was prepared as described previously. Afterwards, the CNT-Silk/BOPP film was cut into a rectangle-shape with dimensions of 15 mm × 35 mm. Then, a small rectangle-shape (2 mm × 30 mm) was cut

off from the middle of the CNT-Silk/BOPP film. Finally, the copper foils were embedded on two ends of the actuator.

**Fabrication of CNT-Silk/BOPP Pressure Sensor**

First, two CNT-Silk films with dimensions of 15 mm × 10 mm were prepared, and copper foils were embedded on the ends of the two films. Then, a BOPP film (10 mm × 10 mm) with a loop structure was cut out and it was sandwiched between two CNT-Silk films to form a sandwich structure. Finally, another two BOPP films were used for encapsulating. The applied voltage was 0.1 V during the sensing performance test.

**Fabrication of the CNT-Silk/BOPP Actuator Integrated with Pressure-Sensing Unit**

The CNT-Silk/BOPP actuator with a pressure adjustment function consists of two parts: the actuating unit and the pressure-sensing unit. First, a U-shaped actuator was prepared according to the previous steps. Second, a copper foil was embedded to one end of the U-shaped CNT-Silk/BOPP actuator. Thrid, a CNT-Silk/BOPP pressure sensor was attached to the other end of the U-shaped actuator as the pressure-sensing unit. Finally, the above materials are encapsulated with BOPP films and a small foam cube was fixed on the CNT-Silk/BOPP pressure-sensing unit.

**Fabrication of Tactile-Activated Gripper**

The tactile-activated gripper consists of three parts. The first part was two U-shaped CNT-Silk/BOPP actuators, and the fabrication method has been described in aforementioned section. The second part was a CNT-Silk/BOPP pressure-sensing unit. The fabrication method was similar to that in aforementioned section. A BOPP film with a loop structure was attached to one end of a U-shaped actuator (attached to the CNT-Silk film side), and a square-shape CNT-Silk film with copper foil was affixed to the BOPP film with a loop structure. The other actuator did not require integrated pressure-pressing unit. Since the two actuators in the gripper were connected in parallel, one pressure-sensing unit can control the magnitude of current in the two actuators simultaneously. The actuator without pressure-sensing unit and

the actuator with pressure-sensing unit were fixed to the outside of a hollow glass cuboid with a BOPP film, and a small foam cube was fixed on the pressure-sensing unit. The third part was an alkaline battery (9 V), which was placed in the hollow glass cuboid. Finally, the circuit was completed according to the circuit diagram (Figure S12c in the Supporting Information).

**Fabrication of Visual Logic Gates**

According to the circuit diagram (Figures S13, S14c, and S15c in the Supporting Information), the pressure-sensing unit was integrated at the end of U-shaped CNT-Silk/BOPP actuator. The fabrication method was similar to that described in the second part of the fabrication of tactile-activated gripper.

**Measurement of Blocking Force**

The CNT-Silk/BOPP actuator was cut into a U-shape with dimensions of 35 mm × 10 mm. One end of the actuator (5 mm) was fixed, while the other end was connected to a preload polydimethylsiloxane (PDMS) cube (33.6 mN). The PDMS cube was placed on a precision balance with an axial configuration. We measured the generation forces of the actuators with different voltages for 5 s.

**Characterization**

The SEM images were captured by a field-emission SEM (Hitachi SU8010). A Raman spectrometer (HORIBA JobinYvon Evolution) was used to record the Raman spectra with the 532 nm He-Ne Laser line. X-ray diffraction (XRD) analysis was studied (Rigaku MiniFlex II) with Cu Kα radiation ($\lambda$= 0.15405 nm). An AC/DC power supply (ZHAOXIN KPS-3005DU, 30V, 5A) was used to provide the AC/DC voltage. Bending angles of the actuators were obtained by reading from optical photos and videos, which were captured by a digital camera (SONY ILCE 6000). The electrical signals were recorded by a digital source meter (Keithley 2410). The temperature was recorded by using a laser sight infrared thermometer (Optris LS).

**Note S2**

In order to investigate the influence of enviromental RH on the actuator, one CNT-Silk/BOPP actuator was prepared and tested under RH of 30%, while the other actuator was prepared and tested under RH of 90%. They were cut into rectangle-shapes with dimensions of 10 mm × 30 mm. Since no electrodes need to be embedded in the actuator, the shape of actuator does not need to be U-shaped. These two actuators were initially placed on a hot plate. They were in flat state when the hot plate was not heated (upper panel of Figure S4, Supporting Information). When the hot plate was heated up to 50 ºC, these two actuators deformed, and the deformation amplitude of them were a little different. As shown in the lower panel of Figure S4 (Supporting Information), when the environmental RH is higher (90%), the deformation of actuator will be larger during the same temperature change. The reason is that when the actuator is fabricated at high RH, the silk absorbs more water and the CNT-Silk film expands more. When the temperature rises, more water content is desorbed from the silk, resulting in a larger shrinkage of the CNT-Silk film. Then, there will be a larger difference in volume change between the CNT-Silk layer and the BOPP layer, which leads to larger actuation performance of the CNT-Silk/BOPP actuator. In a word, the environmental RH has an influence on the actuation performance. Higher RH will lead to larger actuation performance of the CNT-Silk/BOPP actuator.

**Note S3**

Bending curvature calculation principle of the CNT-Silk/BOPP actuator. The parameters are defined as follows (shown in Figure S6 in the Supporting Information):

*L*: The length of the CNT-Silk/BOPP actuator.

*r*: The radius of the arc of the curved CNT-Silk/BOPP actuator.

*x*: The horizontal displacement of the curved CNT-Silk/BOPP actuator.

*y*: The vertical displacement of the curved CNT-Silk/BOPP actuator.

*θ/2*: The chord tangent angle of the curved CNT-Silk/BOPP actuator.

*θ*: The bending angle of the arc of the curved CNT-Silk/BOPP actuator.

The curvature is defined as the reciprocal of radius (1/*r*). The chord tangent angle is given by

$$\frac{\theta}{2} = tan^{-1}\frac{x}{L-y} \qquad \text{(Equation S1)}$$

As the bending angle is given by

$$\theta = \frac{L}{r} \qquad \text{(Equation S2)}$$

The bending curvature 1/*r* is deduced as

$$k = \frac{1}{r} = \frac{\theta}{L} \qquad \text{(Equation S3)}$$

Thus, the bending curvature of the actuator can be calculated by using the bending angle and length of the actuator.

**Note S4**

**OR Gate**

A visual logic gate that performs a function similar to the logic OR gate was also fabricated. As shown in Figure S14a (Supporting Information), the device consists of two input pressure-sensing units (red foam and blue foam), and one output actuating unit. A voltage of 13 V was applied to the device. As shown in Figures 6e and S14b (Supporting Information), when no pressure was applied to red and blue foam cubes, the input signals were “0, 0”, and the circuit was in the “open circuit” state. The actuating unit did not deform, and the output signal was “0”. When the pressure was applied to one of red or blue foam cubes or both of the foam cubes, the input signals were “1, 0”, “0, 1” or “1, 1”, and the circuit was in the “closed circuit” state. The actuating unit was deformed, and the output signal was “1”. The circuit schematic diagrams in different states are shown in Figure S14c (Supporting Information).

**NOT Gate**

Another visual logic gate that implements a function similar to a logic NOT gate was also fabricated. As shown in Figure S15a (Supporting Information), the device consists of one input pressure-sensing unit (red foam) and one output actuating unit. Once an electrical current of 30 mA was applied to the device, the actuating unit deformed. Therefore, as shown in Figures 6g and S15b (Supporting Information), when no pressure was applied to the foam cube on pressure-sensing unit, the input signal was “0”, and the circuit was in the “closed circuit” state. The actuating unit deformed, and the output signal was “1”. When the pressure was applied to the foam cube, the input signal was “1”, and the circuit was in a “short circuit” state. As most current did not pass through the actuating unit, it did not deform and the output signal was “0”. The circuit schematic diagrams in different states are shown in Figure S15c (Supporting Information).

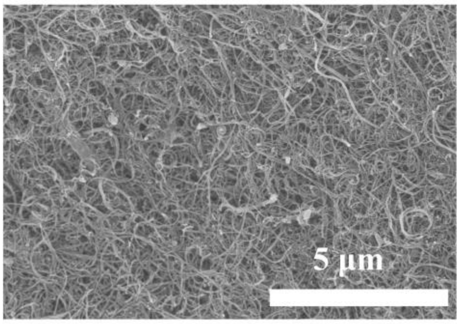


**Figure S1.** SEM image of the surface morphology of the CNT-Silk composite film.

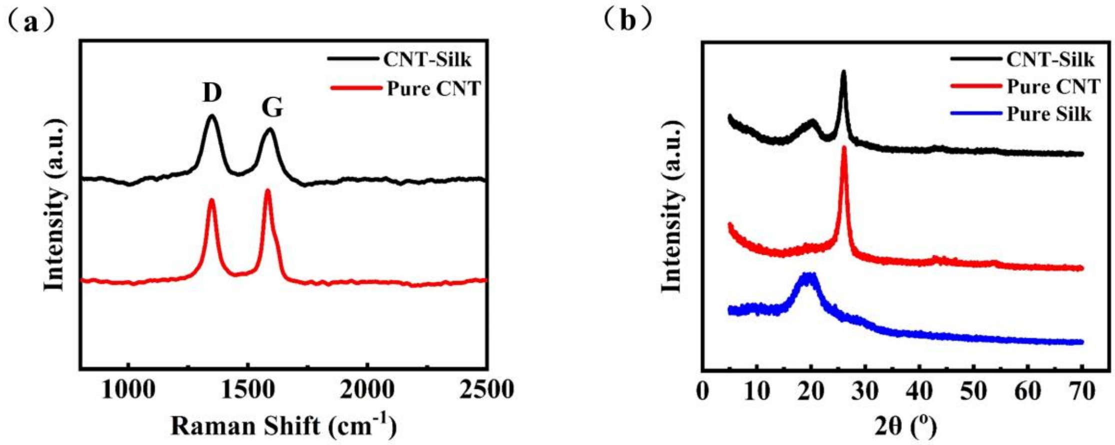


**Figure S2**. (a) Raman spectra of pure CNT and CNT-Silk film. (b) XRD patterns of pure silk fiber, pure CNT, and CNT-Silk film.

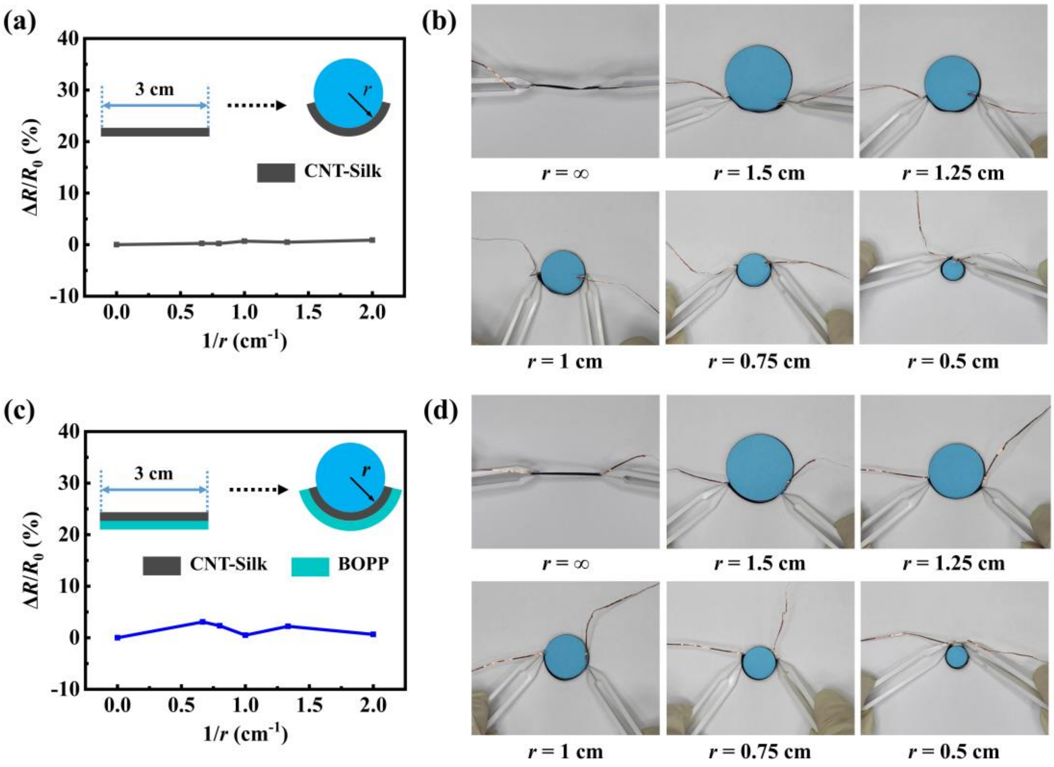


**Figure S3**. (a) Relative resistance change as a function of bending curvature (1/*r*) of the CNT-Silk film. (b) Optical photos of the CNT-Silk film during the bending tests. (c) Relative resistance change as a function of bending curvature (1/*r*) of the CNT-Silk/BOPP film. (d) Optical photos of the CNT-Silk/BOPP film during the bending tests.

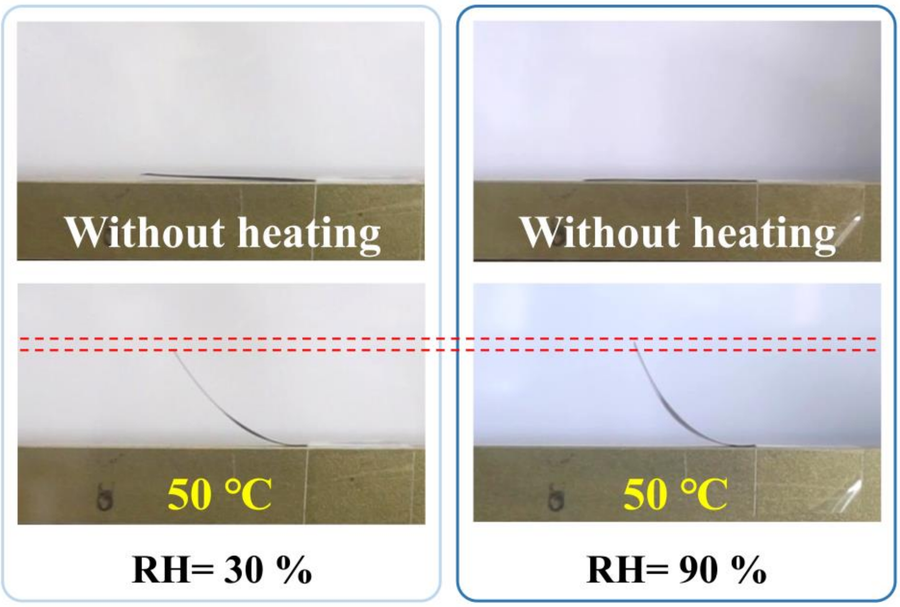


**Figure S4.** Deformation of CNT-Silk/BOPP actuators under RH of 30% (left panel) and under RH of 90% (right panel).

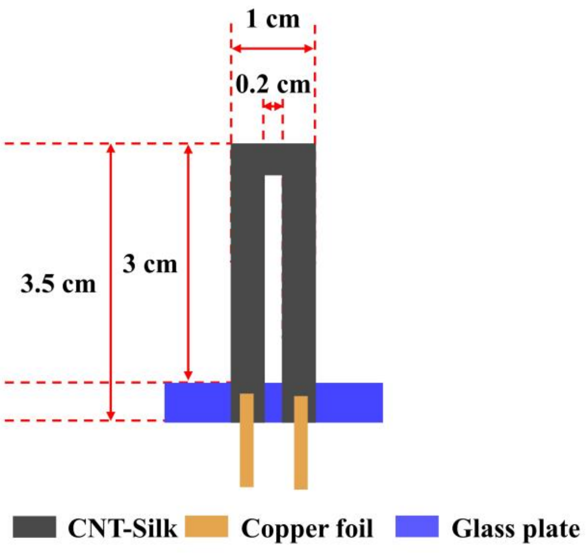


**Figure S5.** Schematic illustration showing the dimensions of CNT-Silk/BOPP actuator.

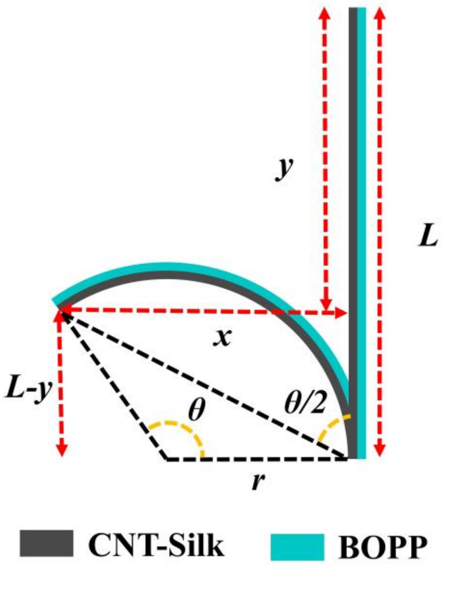


**Figure S6.** Actuator with correlative parameters for calculating the bending curvature.

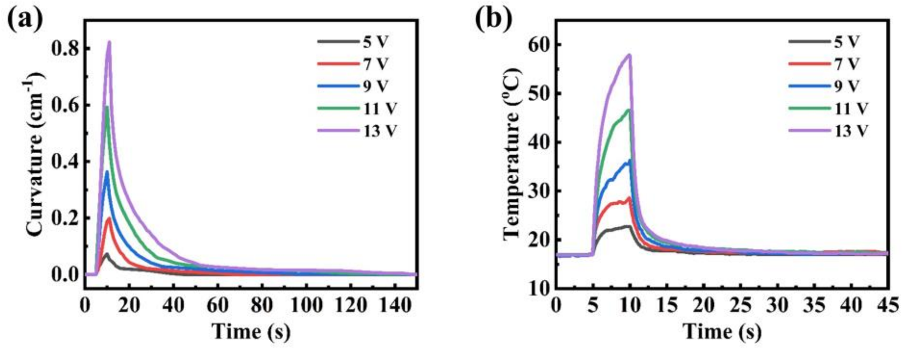


**Figure S7.** (a) Bending curvature of the CNT-Silk/BOPP actuator as a function of time with different driving voltages (5, 7, 9, 11, and 13 V). (b) Temperature of the CNT-Silk/BOPP actuator as a function of time with different driving voltages (5, 7, 9, 11, and 13 V).

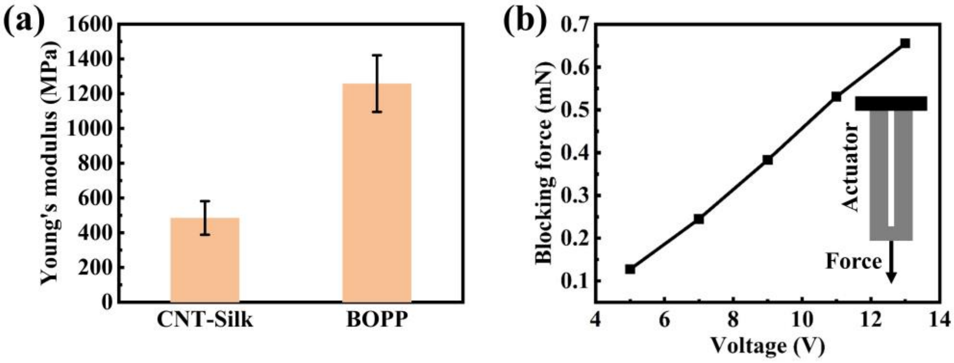


**Figure S8.** (a) Young's moduli of CNT-Silk film and BOPP film. (b) Blocking force of the CNT-Silk/BOPP actuator as a function of driving voltage.

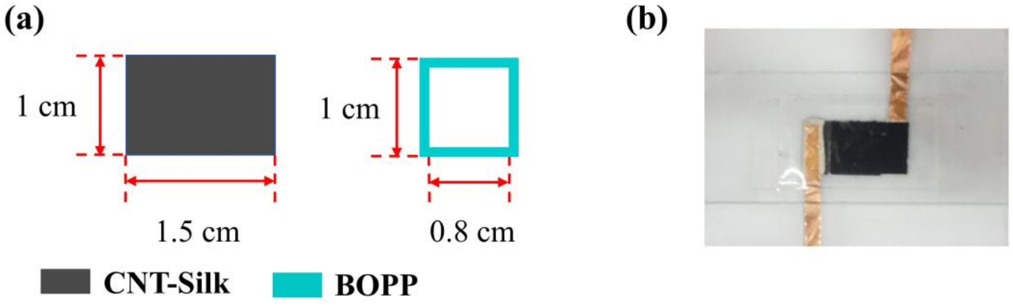


**Figure S9.** (a) Schematic illustration showing the dimensions of CNT-Silk/BOPP pressure sensor. (b) Optical photo of the CNT-Silk/BOPP pressure sensor.

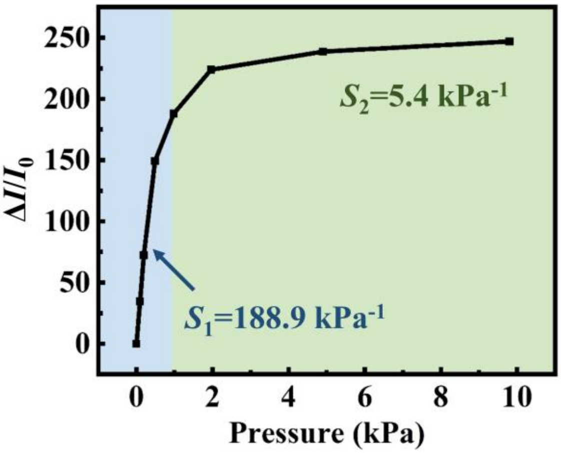


**Figure S10.** Relative current change of the CNT-Silk/BOPP pressure sensor as a function of pressure.

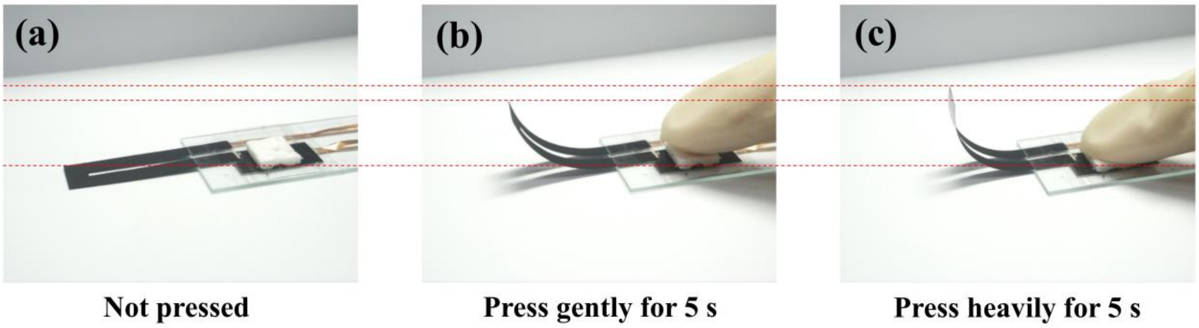


**Figure S11.** CNT-Silk/BOPP actuator integrated with the pressure-sensing unit. The deformation amplitude of the actuator can be controlled by finger pressure. (a) Not pressed. (b) Press gently for 5 s. (c) Press heavily for 5 s.

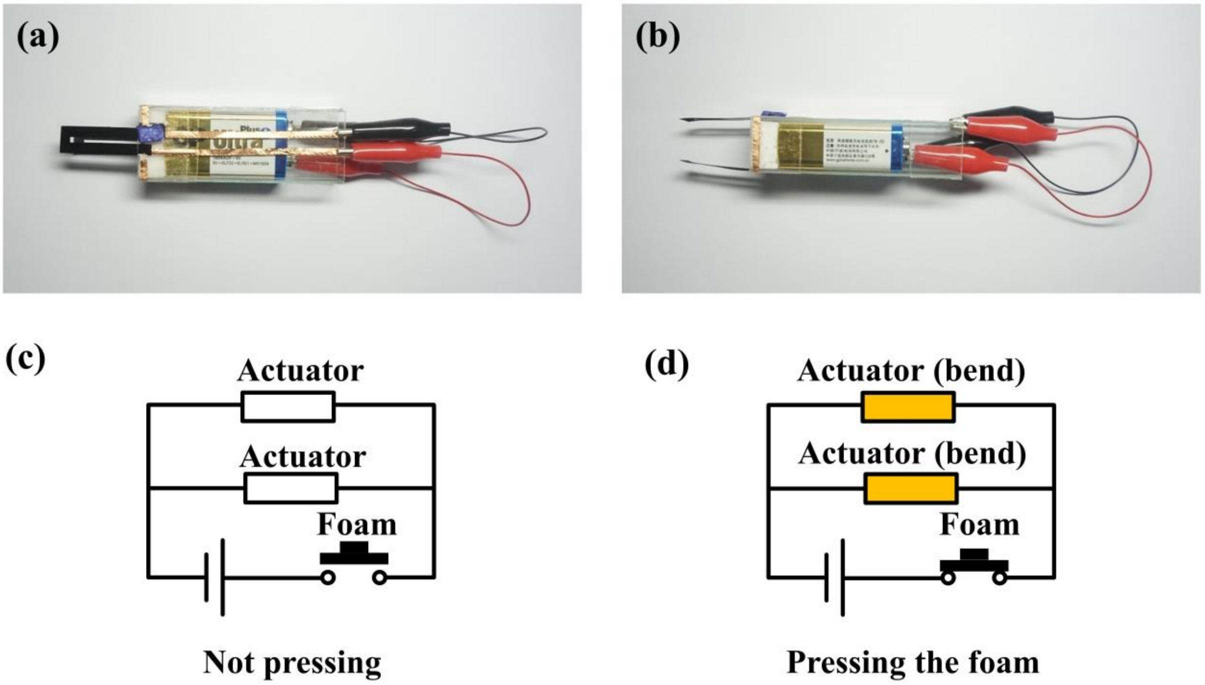


**Figure S12.** Optical photos of the tactile-activated gripper: (a) Top view; (b) Side view. Circuit diagrams for the tactile-activated gripper under different conditions: (c) Not pressing; (d) Pressing the foam cube on pressure-sensing unit.

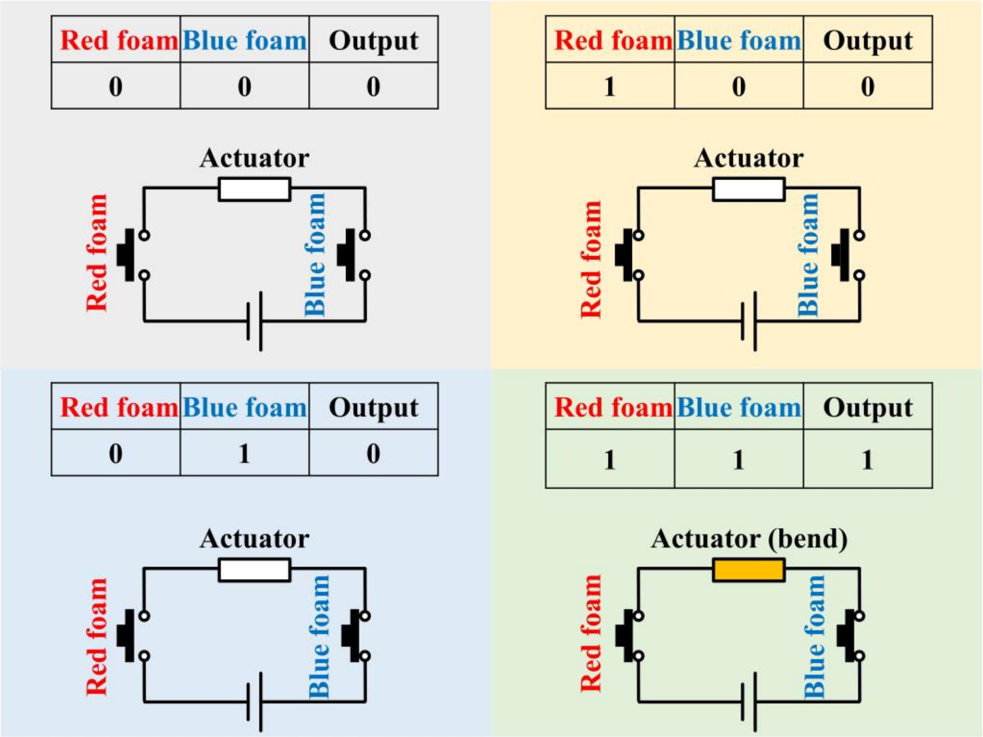


**Figure S13.** Circuit diagrams and truth tables for the visual "AND gate" under different input conditions. Input is the tactile signal. Applying pressure to the foam cube on pressure-sensing unit is defined as input signal "1", and no pressure on the foam cube is defined as input signal "0". Output is the actuating deformation. The deformation of actuating unit is defined as output signal "1", and no deformation of the actuating unit is defined as output signal "0".

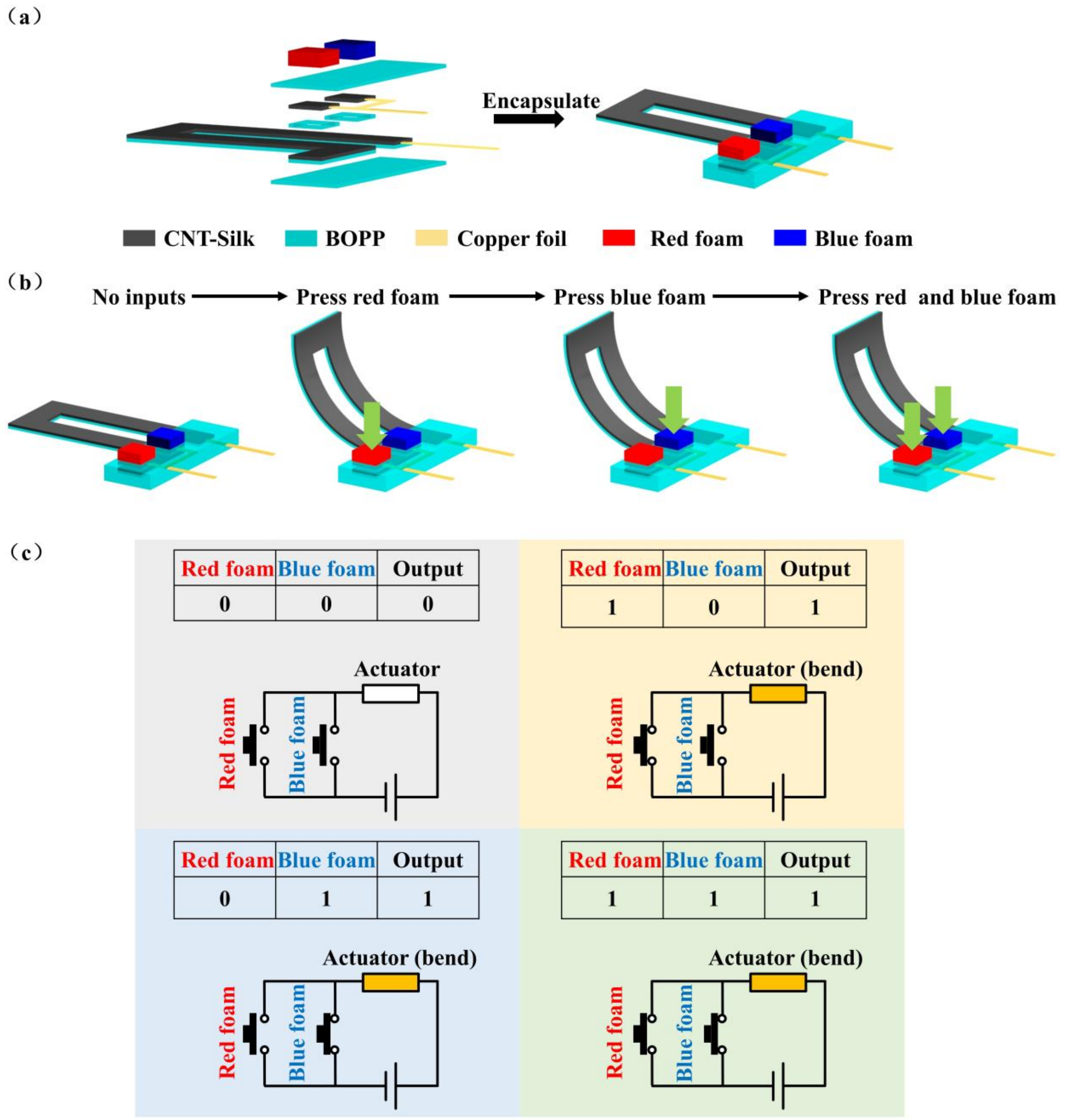


**Figure S14.** (a) Schematic diagram showing the fabrication of visual "OR gate". (b) Schematic diagram showing the mechanism of the visual "OR gate". (c) Circuit diagrams and truth tables for the visual "OR gate" under different input conditions. Input is the tactile signal. Applying pressure to the foam cube on pressure-sensing unit is defined as input signal "1", and no pressure on the foam cube is defined as input signal "0". Output is the actuating deformation. The deformation of actuating unit is defined as output signal "1", and no deformation of the actuating unit is defined as output signal "0".

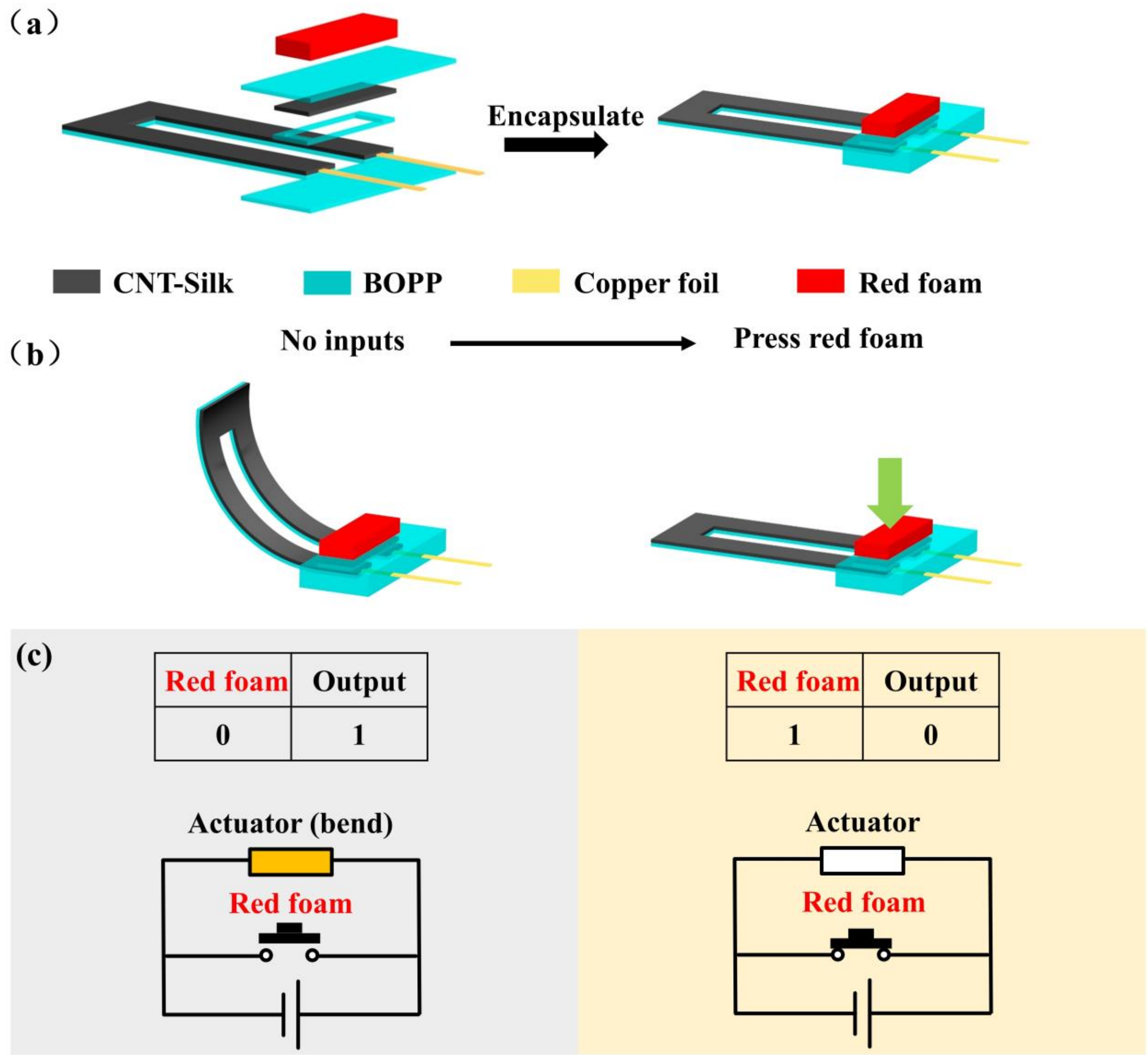


**Figure S15.** (a) Schematic diagram showing the fabrication of visual “NOT gate”. (b) Schematic diagram showing the mechanism of the visual “NOT gate”. (c) Circuit diagrams and truth tables for the visual “NOT gate” under different input conditions. Input is the tactile signal. Applying pressure to the foam cube on pressure-sensing unit is defined as input signal “1”, and no pressure on the foam cube is defined as input signal “0”. Output is the actuating deformation. The deformation of actuating unit is defined as output signal “1”, and no deformation of the actuating unit is defined as output signal “0”.

**Table S1.** Compare the sensitivity of CNT-Silk/BOPP pressure sensor with some recently published CNT-based pressure sensors

| Active Materials | Sensitivity ($kPa^{-1}$) | Detection range (kPa) | Ref. |
|---|---|---|---|
| CNT-Silk | **188.9** | 0 - 9.8 | **Our work** |
| MXene-CNT | 165.4 | 0 - 0.17 | [1] |
| CNT and Ni | 26.13 | 0 - 982 | [2] |
| CNT | 0.515 | 0 - 50 | [3] |
| CNT | 2.72 | 0 - 140 | [4] |
| CNT-Graphene | 0.25 | 0 - 10 | [5] |
| CNT | 0.02 | 0 - 1200 | [6] |
| CNT | 0.127 | 0 - 50 | [7] |
| CNT-Zinc octaethylphorphyrin | 39.4 | 0 - 100 | [8] |
| CNT-Reduced graphene oxide | 4.97 | 0 - 80 | [9] |
| CNT | 1.02 | 0 - 160 | [10] |

**References:**


[1] M. Chen, X. Hu, K. Li, J. Sun, Z. Liu, B. An, X. Zhou, Z. Liu, *Carbon* **2020**, *164*, 111.

[2] S. Pyo, J. Lee, W. Kim, E. Jo, J. Kim, *Adv. Funct. Mater.* **2019**, *29*, 1902484.

[3] L. Miao, J. Wan, Y. Song, H. Guo, H. Chen, X. Cheng, H. Zhang, *ACS Appl. Mater. Inter.* **2019**, *11*, 39219.

[4] C. Wang, X. Hou, M. Cui, J. Yu, X. Fan, J. Qian, J. He, W. Geng, J. Mu, X. Chou, *Sci. China Mater.* **2020**, *63*, 403.

[5] J. Zhai, Y. Zhang, C. Cui, A. Li, W. Wang, R. Guo, W. Qin, E. Ren, H. Xiao, M. Zhou, *ACS Sustain. Chem. Eng.* **2021**, *9*, 14029.

[6] S. Kim, M. Amjadi, T. Lee, Y. Jeong, D. Kwon, M. S. Kim, K. Kim, T. Kim, Y. S. Oh, I. Park, *ACS Appl. Mater. Inter.* **2019**, *11*, 23639.

[7] Z. Qin, X. Sun, Q. Yu, H. Zhang, X. Wu, M. Yao, W. Liu, F. Yao, J. Li, *ACS Appl. Mater. Inter.* **2020**, *12*, 4944.

[8] W. Zhang, Y. Xiao, Y. Duan, N. Li, L. Wu, Y. Lou, H. Wang, Z. Peng, *ACS Appl. Mater. Inter.* **2020**, *12*, 48938.

[9] J. Wu, H. Li, X. Lai, Z. Chen, X. Zeng, *Chem. Eng. J.* **2020**, *386*, 123998.

[10] Y. M. Yin, H. Y. Li, J. Xu, C. Zhang, F. Liang, X. Li, Y. Jiang, J. W. Cao, H. F. Feng, J. N. Mao, L. Qin, Y. F. Kang, G. Zhu, *ACS Appl. Mater. Inter.* **2021**, *13*, 10388.
